# Lycorine Hydrochloride Inhibits the Virulence Traits of* Candida albicans*

**DOI:** 10.1155/2019/1851740

**Published:** 2019-06-03

**Authors:** Longfei Yang, Xin Liu, Yujie Sui, Zhiming Ma, Xuechao Feng, Fang Wang, Tonghui Ma

**Affiliations:** ^1^Jilin Provincial Key Laboratory on Molecular and Chemical Genetics, The Second Hospital of Jilin University, Changchun 130041, China; ^2^Eye Center, The Second Hospital of Jilin University, Changchun 130024, China; ^3^Department of Gastrointestinal Nutrition and Hernia Surgery, The Second Hospital of Jilin University, Changchun 130041, China; ^4^College of Life Science, Northeast Normal University, Changchun 130024, China; ^5^College of Oceanology and Food Science, Quanzhou Normal University, Quanzhou 362000, China

## Abstract

The human opportunistic fungal pathogen* Candida albicans* causes a severe health burden while the biofilms formed by* C. albicans* present a kind of infections that are hard to cure, highlighting the pressing need for new antifungal drugs against* C. albicans*. This study was to explore the antifungal activities of lycorine hydrochloride (LH) against* C. albicans*. The minimal inhibitory concentration (MIC) of LH against* C. albicans* SC5314 was 64 *μ*M. Below its MIC, LH demonstrated antivirulence property by suppressing adhesion, filamentation, biofilm formation, and development, as well as the production of extracellular phospholipase and exopolymeric substances (EPS). The cytotoxicity of LH against mammalian cells was low, with half maximal inhibitory concentrations (IC_50_) above 256 *μ*M. Moreover, LH showed a synergistic effect with AmB, although its interaction with fluconazole, as well as caspofungin, was indifferent. Thus, our study reports the potential use of LH, alone or in combination with current antifungal drugs, to fight* C. albicans* infections.

## 1. Introduction

The opportunistic fungal pathogen* Candida albicans* usually lives in oral cavity, gastrointestinal tract, and urogenital tract and on the skins as a commensal, but when the immune function was compromised, this fungus could cause a series of diseases such as oral thrush, vaginitis, and life-threatening bloodstream infections [1].* C. albicans* cells can undergo the yeast-to-hyphal transition in response to the stimuli encountered in hosts, such as body temperature and epithelial cell contact, to facilitate its survival [2, 3]. The regulation of this transition involves multiple signaling pathways, including Ras1-cAMP-PKA signaling [4, 5]. Hyphae are also critical for tissue invasion and for maintaining the structures of* C. albicans* biofilms, the formation of which starts from adhesion to biotic or abiotic surfaces [6, 7]. Biofilms formed by* C. albicans* on the surfaces of medical devices, such as catheters and prosthetic joints, are often difficult to eradicate, because the condensed cell population and exopolymeric substances (EPS) in biofilms construct a physical barrier, in addition to its elevated expression of drug efflux pumps [7, 8]. Under these circumstances, replacing devices is often necessary, thus imposing a heavy burden on both public health systems and individual patients [8]. The currently used drugs (such as azoles, amphotericin B, and caspofungin) have been associated with resistance, side effects, or low oral bioavailability, while only caspofungin and the lipid formation of Amphotericin B are active against* C. albicans* biofilms, thus making developing new antifungal agents, as well as agents that can improve the efficacy of current antifungal drugs, a pressing mission [9, 10].

**Figure 1:**
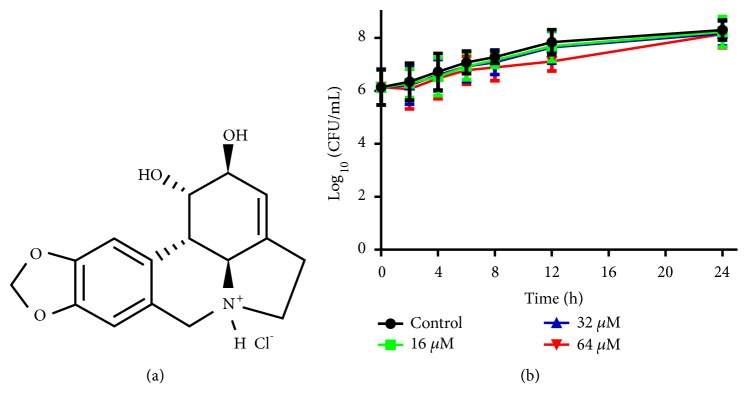
The chemical structure of lycorine hydrochloride (LH) and the Time-killing assay of LH against* C. albicans* SC5314. The initial inoculum of the assay was 10^6^ cells/mL and the incubation was performed at 37°C. *∗* means* p* < 0.05 compared to drug-free controls.

Lycorine hydrochloride (LH, Figure 1(a)) is the major active constituent isolated from the medicinal herb* Lycoris radiata*. It has suppressing effects on the biosynthesis of ascorbic acid in potato tubers [11]. This compound has shown potent antileukemia and antitumor activities against renal cell carcinoma (RCC), ovarian, lung, breast, glioblastoma, melanoma, and esophageal cancer cells at low concentrations through cell cycle arrest and apoptosis induction [12–15]. LH could synergize with cytotoxic T-lymphocyte associated protein 4 (CTLA-4, an immune checkpoint inhibitor) in suppressing RCC in a mouse model [15]. In addition, it could also inhibit the vasculogenic activity of melanoma cells* in vitro* and block the production of blood vessels* in vivo* [16]. What is more important, this compound owns very low toxicity in normal cell lines, as well as in the animal models [12, 13, 16], making it a very promising anticancer candidate.

However, the effects of LH against the human pathogenic fungus* C. albicans* have never been elucidated, although lycorine has been reported to have antifungal activities against* C. albicans* and* C. dubliniensis* [17, 18]. In this study, we first evaluated the antifungal activity of LH against the planktonic cells as well as the biofilms of* C. albicans*. The effects of LH on the virulence factors of* C. albicans* were also investigated.

## 2. Materials and Methods

### 2.1. Chemicals, Strains, and Growth Conditions

LH was bought from National Institutes of Food and Drug Control of China. RPMI-1640 medium powder, 3-(4, 5-dimethyl-2-thiazolyl)-2, 5-diphenyl-2H-tetrazolium bromide (MTT), 2, 3-bis (2-methoxy-4-nitro-5-sulfophenyl)-2H-tetrazolium-5-carboxanilide (XTT), menadione, morpholinepropanesulfonic acid (MOPS), and dibutyryl-cAMP (db-cAMP) were bought from Sigma-Aldrich (Shanghai, China). LH was dissolved in DMSO and stored at -20°C.

*C. albicans* SC5314,* C. albicans* ATCC90028,* Candida glabrata* ATCC2001,* Candida parapsilosis* ATCC22019, and* Candida tropicalis* ATCC7349 bought from China General Microbiological Culture Collection Center (CGMCC) were maintained on yeast extract-peptone-dextrose (YPD) agar medium (1% yeast extract, 2% peptone, 2% dextrose, and 2% agar). Before each test, a colony was picked up and transferred into 5 mL YPD medium in a sterile tube and incubated overnight at 28°C with rotation (140 rpm).

### 2.2. Antifungal Susceptibility Assay

The minimal inhibitory concentrations (MICs) of LH against* Candida* species were determined following microdilution methods from Clinical and Laboratory Standard Institute (CLSI-M27-A3). Overnight grown fungal cultures in YPD medium were collected by centrifugation and diluted to 2 x 10^3^ cells/mL in RPMI-1640 medium (without sodium carbonate, buffered to pH 7.0 with 0.165 M MOPS). 100 *μ*L of such cell suspension was added into each well of 96-well plates. LH was added into each well through serial dilution to achieve various concentrations (4-256 *μ*M). After incubation at 35°C for 24 h, the lowest concentration at which no visual growth was observed was defined as the MIC.

20 *μ*L cell suspension from wells challenged with MIC, 2MIC, 4MIC, and 8MIC of LH was taken and smeared on YPD agar. After incubation at 37°C for 24 h, the colony forming units (CFU) of each well were counted. The minimum fungicidal concentration (MFC) was defined as the lowest concentration at which no colony of fungal strains was grown on the agar plate [19]. The value of MFC divided by MIC was used to judge whether LH had a fungistatic (MFC/MIC > 4) or fungicidal (MFC/MIC < 4) effect [20].

### 2.3. Time-Killing Kinetics

To further confirm the fungicidal or fungistatic effect of LH, time-killing assays were performed. Cell suspensions prepared from overnight YPD cultures were diluted to a density of 10^6^ cells/mL in 1640 medium and 5 mL of such suspension was transferred into each testing tube where* C. albicans* cells were treated with different concentrations of LH. The fungal cells in tubes were grown at 28°C with a rotation of 140 rpm. At 2, 4, 6, 8, 12, and 24 h after the addition of LH, 100 *μ*L cells suspension from each tube was taken out, diluted, and plated on YPD agars. These agars were incubated for 24 h at 37°C before the numbers of CFUs were counted. This assay was performed in triplicate and repeated for three times.

### 2.4. Adhesion Assay

The influence of LH on the adhesion capacity of* C. albicans* on polystyrene surfaces was evaluated by XTT reduction assay, as we described elsewhere [21]. In brief, 100 *μ*L of fungal cell suspension (10^6^ cells/mL in 1640 medium) diluted from overnight YPD culture was transferred into each well of 96-well plates and incubated with 0, 16, 32, and 64 *μ*M of LH at 37°C for 1.5 h. Then, wells were washed with PBS and subjected to XTT assay.

### 2.5. Antibiofilm Assay

The antibiofilm activity of LH against* C. albicans* SC5314 was assessed in 96-well plates [22]. In brief, overnight grown fungal cells in YPD medium were collected by centrifugation (3000g, 5 minutes) and resuspended in RPMI-1640 medium at a density of 10^6^ cells/mL. 100 *μ*L of such suspension was added into wells of 96-well plate. After incubation at 37°C for 24 h, biofilms were formed in each well. Wells containing only medium serve as blank controls.

To test the activity of LH on the biofilm formation, different concentrations of LH were added into wells with cell suspension and plates were incubated for 24 h at 37°C. Free-floating cells in each well were removed by washing with PBS for three times and later the biofilm viability in each well was quantified by XTT reduction assay.

To assess the activity of LH on the preformed biofilms, fresh 1640 medium containing different concentrations of LH was added into each well after 24 h biofilms were washed with PBS. After another incubation for 24 h and PBS washing, XTT reduction assay was performed. These assays were performed in triplicate and repeated for three times.

### 2.6. XTT Reduction Assay

Sterile XTT solution (50 g/L in PBS) was mixed with menadione (final concentration: 1*μ*M) before 100 *μ*L of the mixture was added into each well and incubated in dark for 120 minutes at 37°C. Then, 70 *μ*L of supernatant from each well was transferred into a new 96-well plate and the optical density (OD) at 490 nm of the solution in each well was determined by a microplate reader (VarioSkan, Thermo). The viability of biofilm formed in each well was calculated as viability % = (OD_490nm  treatment_ − OD_490nm  blank_)/(OD_490nm  control_ − OD_490nm  blank_) × 100%, where OD_490nm  treatment_ and OD_490nm  control_ mean OD_490nm_ values of treated biofilms and untreated biofilms while OD_490nm  blank_ is OD_490nm_ values of wells containing only medium without fungal cells [22].

### 2.7. Morphological Transition

To test the influence of LH on the morphological transition of* C. albicans *cells, overnight grown fungal cells were collected and resuspended in three kinds of hyphal-inducing media, namely, RPMI-1640 medium, Spider medium (1% mannitol, 1% nutrient broth, 0.2% K_2_HPO_4_, pH 7.2), and Sabouraud dextrose (SD) medium (4% dextrose, 1% peptone) plus 10% fetal bovine serum (FBS), to achieve a density of 10^6^ cells/mL. The cell suspensions were supplemented with different final concentrations of LH and incubated at 37°C for 4 h statically. Morphological changes in fungal cells were recorded by an inverted microscope (Olympus IX81, Japan).

On the Spider agar (1% mannitol, 1% nutrient broth, 0.2% K_2_HPO_4_, 1.8% agar, pH 7.2),* C. albicans* colonies employ the filamentous growth, and therefore it was used to assess the effects of LH on the morphological changes of fungal colonies [23]. Overnight* C. albicans* cells in YPD medium were collected and resuspended in PBS to get a density of 500 cells/mL. 100 *μ*L of such suspension was plated on Spider agars supplemented with different concentrations of LH and plates were incubated at 37°C for 96 h. Morphologies of colonies were photographed by an anatomical microscope (Olympus SZX16, Japan).

### 2.8. cAMP Rescue Experiment

Db-cAMP was used to investigate the involvement of cAMP in the inhibitory effect of LH on morphological changes. In this assay [23],* C. albicans* cells in 1640 medium (10^6^ cells/mL) were transferred into 96-well plates and cells were exposed to 32 *μ*M LH. Db-cAMP was added into cell suspensions immediately after the addition of LH to achieve a final concentration of 5 mM. Cells treated with the same volume of DMSO or db-cAMP were set as negative controls. After incubation for 4 h at 37°C, morphological changes of cells were recorded by a microscope. These four groups (DMSO, LH, db-cAMP, and LH + db-cAMP) were also used for biofilm formation assay as mentioned above to evaluate the involvement of cAMP in the antibiofilm activity of LH. After treatment for 24 h, the viability of biofilms in each group was determined by XTT reduction assay. This assay was performed in triplicate and repeated for three times.

### 2.9. Phospholipase Production

The influence of LH on the extracellular phospholipase production was evaluated as described elsewhere [24]. In brief, 1 *μ*L of cell suspension (10^6^ cells/mL in 1640 medium) was spotted onto the center of egg yolk emulsion agar supplemented with different concentrations of LH. After 4 days' incubation at 37°C, diameters of colonies (*d*_*1*_) and precipitation zones (*d*_*2*_) surrounding the colonies were measured. The production of phospholipase was expressed in Pz: Pz=*d*_*1*_/*d*_*2*_. This assay was performed in triplicate and repeated for three times.

### 2.10. EPS Production of* C. albicans* Biofilms

The EPS production in preformed biofilms was determined by colorimetry [24]. Preformed biofilms in 24-well plates were challenged by different concentrations of LH for 24 h before biofilms were washed with 0.9% NaCl solution for three times. 0.2 mL NaCl solution and 0.2 mL 0.5% phenol solution were added into each well and mixed well. Then, 2 mL 0.2% hydrazine sulfate solution (in H_2_SO_4_) was slowly added and a two-hour incubation was followed. The OD_490nm_ of the reaction products in each well was measured by a microplate reader.

### 2.11. Checkerboard Assays with Antifungal Drugs

To determine whether LH can synergize with current available antifungal drugs, checkerboard assays were performed as described elsewhere [25]. Fungal cell suspensions were prepared and cultured as described above in antifungal susceptibility tests. The concentrations for AmB and caspofungin acetate were 10 ~ 0.039 *μ*g/mL, and for fluconazole it was 20 ~ 0.078 *μ*g/mL. The concentration for LH was 256 ~ 4 *μ*M. After 24 h incubation at 37°C, MIC of each drug was recorded to calculate the fractional inhibitory concentration (FIC). FICI = FICI_A_ + FICI_B_ = MIC_A  combination_/MIC_A  alone_ + MIC_B  combination_/MIC_B  alone_. The combination is considered synergistic when FICI ≤ 0.5, indifferent when FICI > 0.5 and ≤ 4, and antagonist when FICI > 4.

### 2.12. Cytotoxicity against Human Cells

MTT assays performed on HUVEC and Chang's liver cells were used to assess the cytotoxicity of LH, as previously described elsewhere [26].

### 2.13. Statistical Analysis

Each assay was performed in triplicate and repeated for at least three times. Graphs shown were produced by GraphPad Prism Software while data were presented as means with standard deviation (SD). Student's* t*-tests were performed to calculate the differences between treatment and drug-free controls. *∗*,* p*<0.05.

## 3. Results

In the present study, we first examined the antifungal activity of LH against the five* Candida* strains belonging to four species according to CLSI guidelines. The MICs and MFCs were listed in Table 1. The MICs of LH against two* C. albicans* strains were 64 *μ*M while the MFCs were above 256 *μ*M (4MIC), as there were still colonies grown on the solid agar after fungal cells were exposed to this concentration for 24 h. Therefore, the MFC/MIC value is above 4, indicating a fungistatic effect. Due to the prevalence of* C. albicans* and the well-known genetic background of* C. albicans* SC5314, this strain was selected for further research. To further confirm the fungistatic effect of LH, the time-killing assay was performed. As shown in Figure 1(b), the concentration used (16-64 *μ*M) did not decrease the viable cells in this assay, consistent with the fungistatic effect of LH revealed by the high value of MFC/MIC.

**Figure 2:**
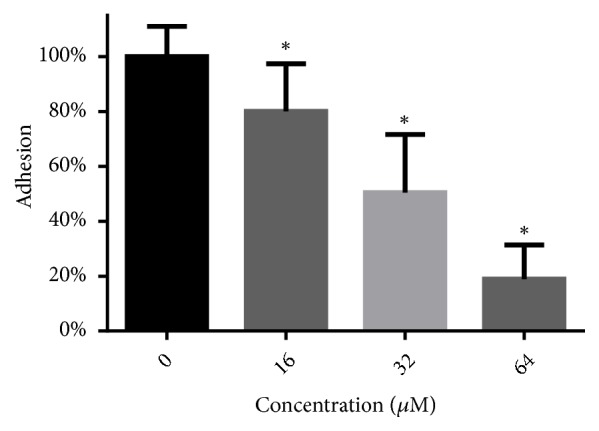
LH decreases the adhesion capacity of* C. albicans* to polystyrene surfaces. After treatment with LH for 1.5 hours at 37°C, XTT assays were performed to calculate the viability of cells left on polystyrene surfaces after PBS washing. *∗* means* p* < 0.05 compared to drug-free controls.

As shown in Figure 2, 16-64 *μ*M of LH significantly decreased the adhesion of* C. albicans* cells to polystyrene surfaces of microplates. At the highest concentration used (64 *μ*M), LH could decrease about 80% of adhesion, as compared to drug-free controls.

**Figure 3:**
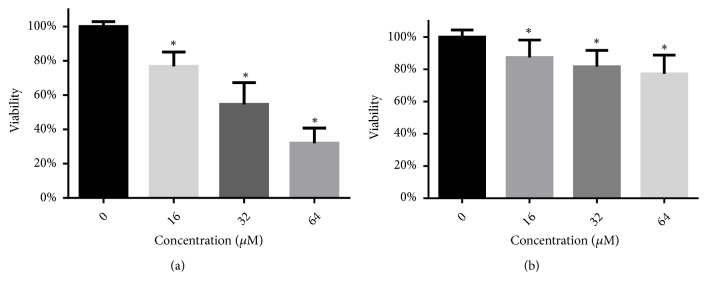
The effects of LH on the formation and development of* C. albicans* biofilm. (a) After incubation with different concentrations of LH under biofilm forming conditions for 24 h, the viability of biofilms in 96-well plates was determined by XTT reduction assay. (b) Preformed biofilms were further cultured for 24 h in the presence of LH, followed by XTT assay. *∗* means* p* < 0.05 compared to drug-free controls.

**Figure 4:**
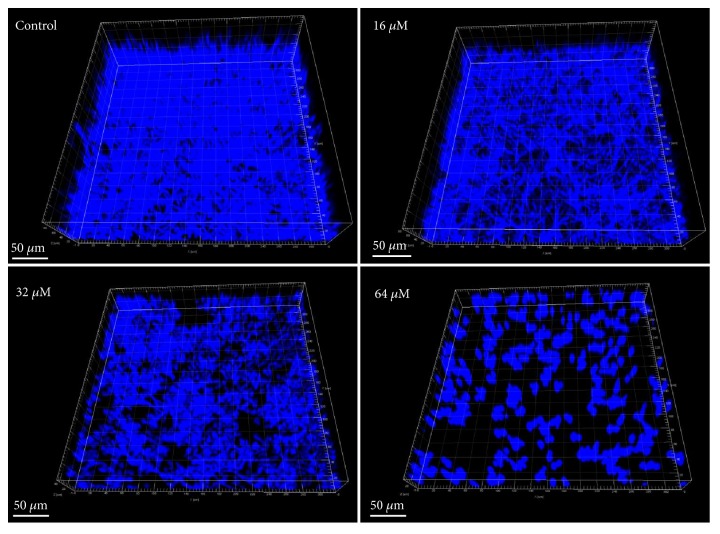
The effects of LH on the formation of* C. albicans* biofilms. The 3D structures of* C. albicans* biofilms were reconstructed by Imaris 7.02 using z-axis photos recorded by confocal microscope.

The antibiofilm activity of LH was quantified through XTT reduction assay. In the biofilm formation assay (Figure 3(a)), treatment with 16-64 *μ*M of LH could significantly reduce the metabolic viability of* C. albicans* cells in biofilms. The antibiofilm activity of LH could also be seen in Figure 4, where the 3D structures of* C. albicans* biofilms were visualized by Imaris software using* z*-axis photographs recorded by confocal microscope. As shown in Figure 4, increasing the LH concentration would result in fewer hyphae and total* C. albicans* cells. As for preformed biofilms, exposure to 16-64 *μ*M of LH could only reduce the viability of mature biofilms by 20%-30% (Figure 3(b)), as compared to drug-free controls.

**Figure 5:**
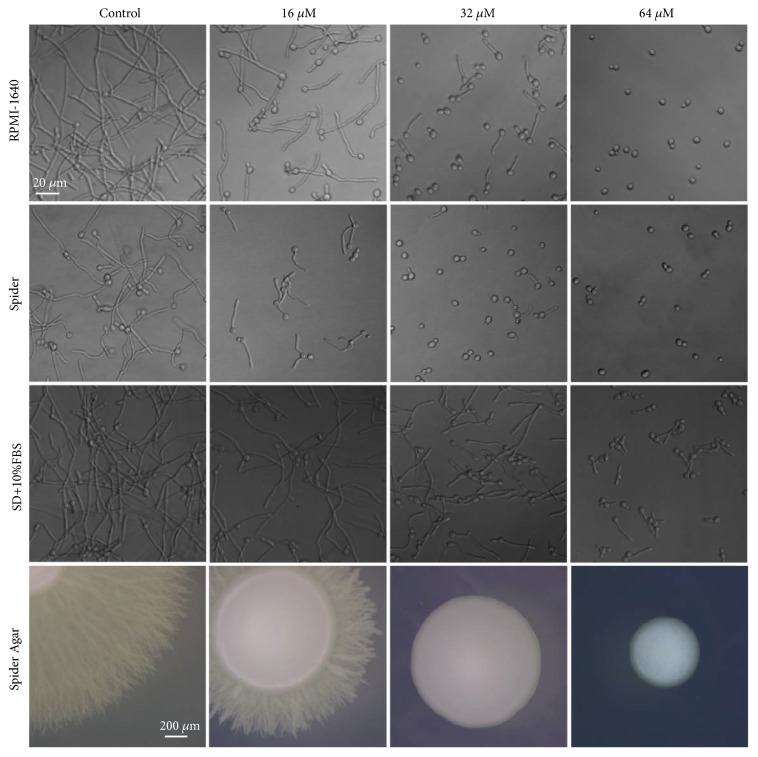
LH inhibits the yeast-to-hyphal transition of* C. albicans*. 10^6^ cells/mL* C. albicans* with various concentrations (0, 16, 32, and 64 *μ*M) of LH in RPMI-1640 medium, spider medium, or SD broth supplemented with 10% FBS were incubated at 37°C for 4 h and recorded by an inverted microscope. Magnification, 40×. Morphologies of colonies on Spider agars were photographed by an anatomical microscope, after incubation at 37°C for 96 h.

To test the inhibitory effects of LH on filamentation in response to different stimuli (neutral pH and nutrition limitation), 1640 medium and Spider medium were used to induce hyphal formation. As expected, LH could suppress the hyphal formation in a concentration-dependent manner in both media tested (Figure 5). We also investigated the inhibitory capacity of LH on filamentation in the presence of serum, which is a strong inducer and is a key requirement for filamentation in the host. Although a little weaker inhibitory activity was displayed, LH did suppress the filamentation of* C. albicans* induced by serum (Figure 5). The filamentous growth on Spider agar could also be suppressed by LH, evidenced by shortened peripheral filaments around the central smooth colony in the presence of LH.

**Figure 6:**
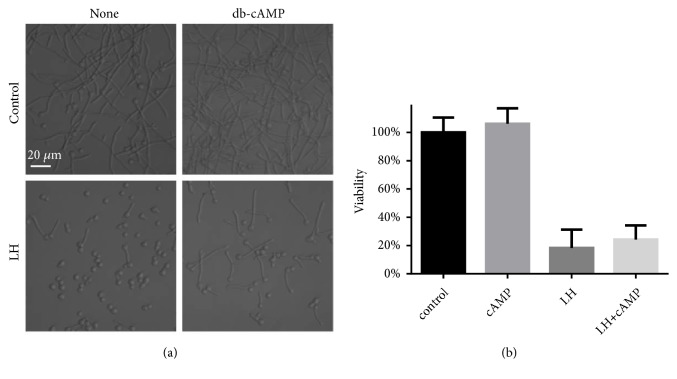
cAMP is involved in the inhibitory effects of LH on hyphal induction and biofilm formation of* C. albicans*. (a) The inhibitory effect of LH on hyphal formation could be rescued by addition of cAMP analog. (b) Treatment with db-cAMP saved part of cell viability of cells in biofilms challenged by LH.

In the hyphal growth, cAMP plays an important role. Thus, we tested whether cAMP was involved in the hyphal inhibition by LH. As shown in Figure 6(a), the addition of exogenous cAMP analog, namely, db-cAMP, could rescue the hyphal formation inhibition induced by LH exposure. This indicated that LH inhibits hyphal formation through decreasing cAMP in* C. albicans* cells. The effects of exogenous cAMP on the biofilm inhibition caused by LH were also tested, through XTT assay. As revealed by Figure 6(b), although the addition of 5 mM db-cAMP could rescue part of biofilm viability, the differences were not statistically significant.

**Figure 7:**
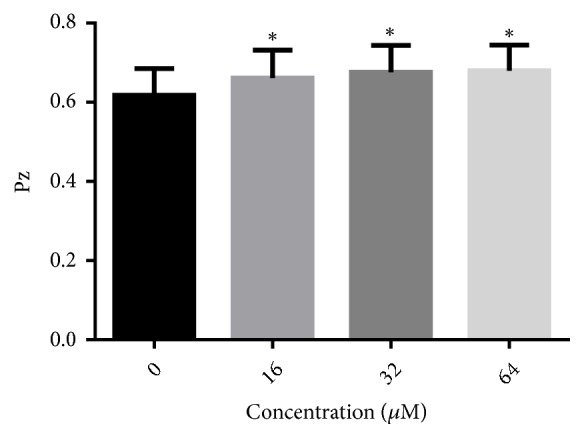
The effect of LH on the production of extracellular phospholipase of* C. albicans*. 1 *μ*L of cell suspension (1x10^6^ cells/mL) was spotted onto phospholipase agar and incubated at 37°C for 4 days. Pz value means the ratio of the diameter of colony to the diameter of colony plus the precipitation zone. The smaller Pz value indicates the stronger phospholipase activity. *∗* means* p* < 0.05 compared to drug-free controls.

Extracellular phospholipase production of* C. albicans* could also be decreased by treatment with LH, as evaluated by the egg yolk emulsion method. As shown in Figure 7, 16-64 *μ*M of LH increased the Pz value significantly, in a concentration-dependent way.

**Figure 8:**
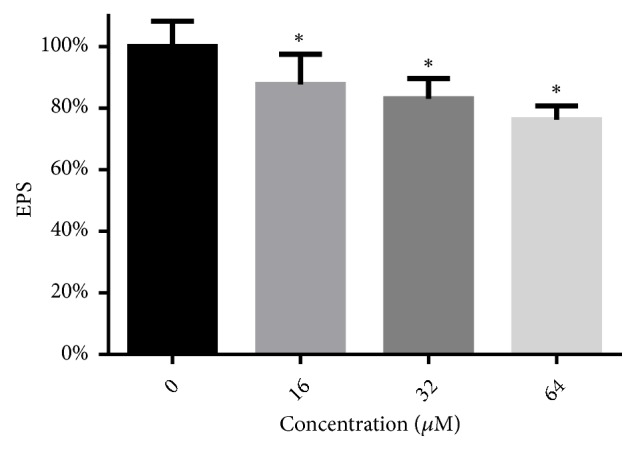
LH inhibits the production of EPS in* C. albicans* biofilms. EPS production in preformed biofilms was determined by the phenol-sulfuric acid method. *∗* means* p* < 0.05 compared to drug-free controls.

EPS of preformed biofilm represents a physical barrier that prevents the access of antifungal drugs into cells within biofilms. The influence of LH on the EPS production in preformed* C. albicans* biofilms was evaluated by determining the OD_490nm_ of the reaction products of EPS and hydrazine sulfate/phenol. As shown in Figure 8, preformed biofilms treated with 16-64 *μ*M of LH produced less EPS, as compared to drug-free controls. This inhibition was also dose-dependent.

To test whether LH can synergize with current available antifungal drugs, checkerboard assays were performed. As shown in Table 2, CAS and FLZ have no interactions with LH; that is to say, at least LH cannot impair or attenuate the efficacy of CAS and FLZ, if used together. A synergistic effect was observed between the combinations of LH and AmB.

The cytotoxicity of LH against mammalian cells was evaluated through proliferation inhibition assays. The half maximal inhibitory concentrations (IC_50_) of LH against both Chang's liver cells and HUVEC cells were above 256 *μ*M (Table 3), indicative of its low cytotoxicity.

## 4. Discussion

*C. albicans* represents a major fungal pathogen, causing several kinds of infections including oral thrush, vaginitis, and candidemia [1]. The imperfect pharmacological properties of current antifungal drugs, along with resistance, make it a necessary and pressing mission to develop new antifungal agents, especially those effective against* C. albicans* biofilms, which are notorious in clinic context for its resistance and recalcitrance, and cause a heavy burden on patients with catheters [27]. Natural products, especially those from traditional medicinal herbs, represent a potential reservoir for mining antifungal agents [26–28]. LH, separated from the traditional Chinese herb* Lycoris radiata *which has been used for hundreds of years, has been reported to have antitumor activity against multiple kinds of cancer cell lines [12, 14–16, 29]. In this study, we for the first time report the* in vitro* antifungal activity of LH against the human fungal pathogen* C. albicans*.

The MICs of LH against* C. albicans* SC5314 and ATCC 10231 were both 64 *μ*M, which was much lower than that of lycorine against* C. albicans* (about 200 *μ*M) and* C. dublinensis* (about 100 *μ*M) [17, 18]. The fungistatic effect of LH could be inferred from high MFC/MIC ratio (above 4), as well as nonreduction curves in the time-kill assays where a reduction of more than 3 log_10_ (CFU/mL) relative to initial inoculum was considered as fungicidal activity [24, 26]. Therefore, LH did exert a fungistatic effect.

Biofilms of* C. albicans* often represent a notorious life style that undermines the therapeutic efficacy of current antifungal drugs. Thus, we assessed the activity of LH against* C. albicans* biofilm, in both formation and development phases. As revealed by XTT assays, LH could inhibit the formation and development of* C. albicans* biofilms. The inhibition on biofilm formation could also be confirmed by CLSI images. The inhibition on preformed biofilms was weaker than that on biofilm formation, which can also be seen with many other antifungal agents [21, 24, 26]. This may be due to the condensed networks of intertwined hyphae and the existence of extracellular matrix, which compromise the access of drugs into cells within biofilms. As for the EPS, one of the major components of the extracellular matrix, LH could also decrease its production in preformed biofilms, and the extent of inhibition was similar to and consistent with its inhibition on the viability of preformed biofilms. This further indicates the potential of LH to combat* C. albicans* infections. In addition, adhesion, as the first move of* C. albicans* to form biofilm on biotic or abiotic surfaces, was also suppressed by treatment with LH. This suppression may be associated with decreased expression of adhesins, such as Hwp1 and Als3, which need to be further confirmed [30, 31].

Hyphal formation in* C. albicans* is closely associated with biofilm formation and other virulence factors, and mutants defective in hyphal formation simultaneously demonstrated defects in biofilm formation [32]. Hyphal cells can produce the cytolytic peptide toxin, candidalysin, to cause damage on mucosa [33]. Moreover, hyphal-specific Als3 of* C. albicans* can promote the iron acquisition from ferritin, thus compromising host nutritional immunity [3, 34]. Also, during hyphal growth, the expressions of PRA1 and ZRT1 genes are increased to promote zinc acquisition in milieu [35]. Therefore, the hyphal growth of* C. albicans* may represent a novel target to develop antivirulence strategies to fight* C. albicans* infections [36, 37]. Hyphal formation in different media is induced by different stimuli that were encountered by* C. albicans* and is mediated by multiple filamentation-inducing pathways. For example, hyphal growth in Spider medium is mediated by cAMP, GlcNAc medium by Efg1, and 1640 medium by neutral pH [36, 38]. Our data showing that LH inhibited hyphal growth in different media indicated its inhibition on diverse hyphal-inducing signaling pathways.

Extracellular phospholipase of* C. albicans*, the expression of which was increased during* C. albicans* infections, can break down lipids of membrane for tissue invasion, while* C. albicans* mutants with phospholipase defects showed attenuated virulence in murine infection models [21, 24, 39, 40]. Our results showed that LH could inhibit the production of extracellular phospholipase. This inhibition on phospholipase production, which could also be demonstrated by other antifungal agents [41, 42], may further contribute to its antifungal effects.

Fungal cells and human cells are both eukaryotes, resulting in the paucity of targets that could be employed for treating fungal infections. The limited antifungal drugs were impeded by unwanted side effects and the development of drug resistance, which further contributes to the severe status of fungal infections. With the deeper understanding of the pathogenesis of* Candida* infections, it was increasingly accepted that targeting virulence factors without causing cell death might be an attractive strategy [36, 39, 43]. There are already published researches on compounds which inhibit virulence factors rather than induce death of fungal cells [36]. These compounds have demonstrated therapeutic efficacy* in vivo*, corroborating the plausibility of this antivirulence strategy.

Combination therapy can lower the side effects associated with high doses of antifungal drugs and impose less selection pressure for the antifungal drug resistance [44, 45]. Although LH did not show synergistic effects with FLZ and CAS, it did show synergistic activity when combined with AmB. In other words, the use of LH could help to lower the dose of AmB, thus mitigating the toxicity of AmB.

Although LH has demonstrated potent activities of growth inhibition against ovarian cancer cells (IC_50_ = 1.2 *μ*M) [12], the inhibitory effects against Chang's liver cells and HUVEC cells were weak, with IC_50_ higher than 256 *μ*M. These results confirmed the selectivity of LH, and so was the safety, which could also be seen in mice and dogs receiving daily injection: LH did not cause obvious effects, such as loss of body weight [12, 13]. The low toxicity, combined with its inhibitory activity against virulence factors, suggests that LH is a promising candidate for anti-*Candida* therapy development, although the* in vivo* antifungal activity of LH and the underlying mechanisms remain to be elucidated.

## 5. Conclusion

In summary, the fungistatic LH is effective against* C. albicans*, in both planktonic and biofilm forms. LH could also inhibit the virulence traits such as filamentous growth, adhesion, production of extracellular degrading enzymes, and EPS, which play important roles in the pathogenicity of* C*.* albicans*. Moreover, LH could synergize with AmB in inhibiting* C. albicans*. Since LH did not exert fungicidal activity against* C. albicans*, it may confer less selective pressure for incurring resistance [46]. Our current study may provide a promising candidate for developing antifungal therapies, although much more research on the* in vivo* activity and pharmacokinetics of LH is needed before its entry into clinic use.

## Figures and Tables

**Table 1:** The antifungal activities of LH against *Candida species*.

Fungal strains	MIC (*μ*M)	MFC (*μ*M)
*C. albicans* SC5314	64	>512
*C. albicans* ATCC10231	64	512
*C. glabrata* ATCC2001	64	512
*C. parapsilosis* ATCC22019	64	>512
*C. tropicalis* ATCC7349	128	>512

**Table 2:** Interaction of LH with antifungal drugs.

Drug A	MIC of drug A (*μ*g/mL)		MIC of LH (*μ*M)		FICI	Interaction
	Alone	Combined	Alone	Combined		
AmB	1.25	0.3125	64	16	0.5	Synergistic
CAS	0.625	0.3125	64	64	1.5	Indifferent
FLZ	1.25	0.625	64	64	1.5	Indifferent

**Table 3:** Cytotoxicity of LH against mammalian cell lines.

Cell lines	IC_50_ (*μ*M)
Chang's liver cells	>256
HUVEC	>256

## Data Availability

The data used to support the findings of this study are available from the corresponding author upon request.
