# Corrigendum to “Lycorine Hydrochloride Inhibits the Virulence Traits of Candida albicans”

**DOI:** 10.1155/2021/1294536

**Published:** 2021-01-02

**Authors:** Longfei Yang, Xin Liu, Yujie Sui, Zhiming Ma, Xuechao Feng, Fang Wang, Tonghui Ma

**Affiliations:** ^1^Jilin Provincial Key Laboratory on Molecular and Chemical Genetics, The Second Hospital of Jilin University, Changchun 130041, China; ^2^Eye Center, The Second Hospital of Jilin University, Changchun 130024, China; ^3^Department of Gastrointestinal Nutrition and Hernia Surgery, The Second Hospital of Jilin University, Changchun 130041, China; ^4^College of Life Science, Northeast Normal University, Changchun 130024, China; ^5^College of Oceanology and Food Science, Quanzhou Normal University, Quanzhou 362000, China

In the article titled “Lycorine Hydrochloride Inhibits the Virulence Traits of Candida albicans” [1], there are minor text changes required in Sections “2.1. Chemicals, Strains, and Growth Conditions” and “2.2. Antifungal Susceptibility Assay” of the article body. “ATCC90028” should be corrected to “ATCC10231” and “(MFC/MIC > 4)” should be corrected to “(MFC/MIC ≥ 4)”. The corrected content of Sections 2.1 and 2.2 is shown below:

## 2. Materials and Methods

### 2.1. Chemicals, Strains, and Growth Conditions

LH was bought from National Institutes of Food and Drug Control of China. RPMI-1640 medium powder, 3-(4, 5-dimethyl-2-thiazolyl)-2, 5-diphenyl-2H-tetrazolium bromide (MTT), 2, 3-bis (2-methoxy-4-nitro-5-sulfophenyl)-2H-tetrazolium-5-carboxanilide (XTT), menadione, morpholinepropanesulfonic acid (MOPS), and dibutyryl-cAMP (db-cAMP) were bought from Sigma-Aldrich (Shanghai, China). LH was dissolved in DMSO and stored at -20°C.

C. albicans SC5314, C. albicans ATCC10231, Candida glabrata ATCC2001, Candida parapsilosis ATCC22019, and Candida tropicalis ATCC7349 bought from China General Microbiological Culture Collection Center (CGMCC) were maintained on yeast extract-peptone-dextrose (YPD) agar medium (1% yeast extract, 2% peptone, 2% dextrose, and 2% agar). Before each test, a colony was picked up and transferred into 5 mL YPD medium in a sterile tube and incubated overnight at 28°C with rotation (140 rpm).

### 2.2. Antifungal Susceptibility Assay

The minimal inhibitory concentrations (MICs) of LH against Candida species were determined following microdilution methods from Clinical and Laboratory Standard Institute (CLSI-M27-A3). Overnight grown fungal cultures in YPD medium were collected by centrifugation and diluted to 2 × 103 cells/mL in RPMI-1640 medium (without sodium carbonate, buffered to pH 7.0 with 0.165 M MOPS). 100 *μ*L of such cell suspension was added into each well of 96-well plates. LH was added into each well through serial dilution to achieve various concentrations (4-256 *μ*M). After incubation at 35°C for 24 h, the lowest concentration at which no visual growth was observed was defined as the MIC.

20 *μ*L cell suspension from wells challenged with MIC, 2MIC, 4MIC, and 8MIC of LH was taken and smeared on YPD agar. After incubation at 37°C for 24 h, the colony-forming units (CFU) of each well were counted. The minimum fungicidal concentration (MFC) was defined as the lowest concentration at which no colony of fungal strains was grown on the agar plate [19]. The value of MFC divided by MIC was used to judge whether LH had a fungistatic (MFC/MIC ≥ 4) or fungicidal (MFC/MIC < 4) effect [20].
